# How much do field mice prefer dwarf bamboo seeds? Two‐choice experiments between seeds of *Sasa borealis* and several tree species on the forest floor

**DOI:** 10.1002/ece3.10636

**Published:** 2023-10-19

**Authors:** Hanami Suzuki, Hisashi Kajimura

**Affiliations:** ^1^ Laboratory of Forest Protection, Graduate School of Bioagricultural Sciences Nagoya University Nagoya Japan

**Keywords:** acorn, *Apodemus argenteus*, *Apodemus speciosus*, masting, *Sasa borealis*, seed preference

## Abstract

Bambusoideae is a taxon of mass‐flowering monocarpic perennials with a long life cycle. Forest ecosystems are affected by Bambusoideae seeding and death events in various ways, including an increased abundance of *Apodemus* spp. The utilization and preference of dwarf bamboo seeds over tree seeds by field mice remain elusive. Therefore, we aimed to determine whether field mice prefer dwarf bamboo to tree seeds. We examined one dwarf bamboo species (*Sasa borealis*) against four tree species with varying acorn/fruit traits (*Castanea crenata*, *Quercus crispula*, *Fagus crenata*, and *Lindera triloba*). The seeds were placed in a container in a forest among dead *S. borealis* culms, with an automatic camera monitoring the setup. The examined seeds were mainly foraged by two field mouse species, *Apodemus speciosus* and *Apodemus argenteus,* with preference in the following order: *C. crenata*, *L. triloba*, *S. borealis*, *F. crenata*, and *Q. crispula*. Our findings indicated that during *S. borealis* mast seeding years, predation pressure on *F. crenata* and *Q. crispula* seeds could be considerably reduced. This suggests that mast seeding might disrupt the normal pattern of survival, and seed dispersal patterns, potentially altering the forest vegetation composition.

## Graphical abstract

In this study, we evaluate the preference of field mice (*Apodemus speciosus* and *A. argenteus*) for dwarf bamboo (*Sasa borealis*) seeds, which are supplied once every 120 years by a mast seeding event, in comparison with seeds of four tree species in the forest ecosystem. The key results show a higher preference for *S. borealis* seeds than for two of the tree species. This finding indicates that *S. borealis* mast seeding can disrupt the normal pattern of survival and dispersal of tree seeds and may eventually impact the vegetation composition of forests.

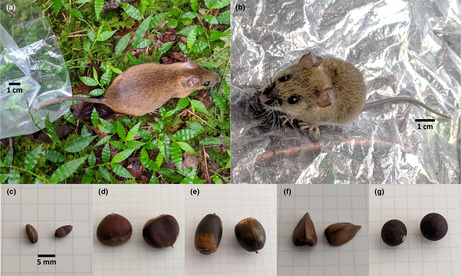

## 1 INTRODUCTION

Bambusoideae, including bamboo and dwarf bamboo, exhibit large‐scale flowering, seeding, and death (hereinafter referred to as “mast seeding”), in a cycle of three to over 120 years (Janzen, 1976). For example, the dwarf bamboo species *Sasa borealis* (Hack.) Makino et Shibata undergoes a flowering cycle every 120 years. This flowering is often reported to be synchronized intraspecifically (e.g., Cho et al., 2017; Nagata & Tamura, 2014; Niiyama et al., 2021). Such synchronization factor is evolutionarily grounded in the principle of predator satiation, where producing many seeds simultaneously increases the probability of escaping predators (Janzen, 1971). *S. borealis* generally germinates 2 years after seeding, marking the initiation of the subsequent generation (Nakagawa et al., 2023).

The dwarf bamboo, a type of Bambusoideae that covers the forest floor, provides a habitat for field mice. These mice prefer dense forest floors for foraging and as an escape zone to protect themselves from predators (Sakamoto et al., 2012; Teixeira et al., 2017; Wada, 1993). Its evergreen leaves are a major food resource for sika deer (*Cervus nippon* [Temminck, 1838]), particularly during winter (Koizumi et al., 2006; Takatsuki, 1983; Tanaka et al., 2008). Dwarf bamboo is also considered a strong inhibitor of forest regeneration due to its sunlight‐blocking dense and long‐term growth (Doležal et al., 2009; Hasegawa & Fujibe, 2015; Nakashizuka & Numata, 1982; Uno et al., 2019). These characteristics suggest that bamboo is an important component of forest ecology, implying that the impact of a bamboo mast seeding event would be significant.

One of the impacts of a bamboo mast seeding event is a large seed supply on the forest floor. Bamboo seeds are considered highly valuable because their crystalline morphology and starch structure are comparable to rice, although the content is slightly lower (Ai et al., 2016). Historically, bamboo seeds were used as food by humans (Kiruba et al., 2007). Birds and rodents have been reported consuming bamboo seeds in the wild (Areta et al., 2009; Areta & Cockle, 2012; Franklin, 2005; Kitzberger et al., 2007; Lebbin, 2006). In particular, the mast seeding of Bambusoideae leads to a rapid outbreak of rodent populations (Bovendorp et al., 2020; González et al., 2000; Ito, 1975; Suzuki et al., 2022). Nevertheless, studies on rodents' preferences for bamboo seeds over other seeds are few, with only one reported from Argentina by Kitzberger et al. (2007). They conducted experiments with seeds of higher and lower food quality than bamboo seeds and found that the presence of bamboo seeds altered the predation pressure on seeds of varying qualities. Forest rodents are not only seed predators but also seed dispersers owing to their seed‐caching habits (Klinger & Rejmanek, 2009). Therefore, understanding how the abundant bamboo seed supplied by mast seeding alters the food selection of rodents and its potential effects on other plants in the forest ecosystem is essential.

Factors influencing rodent seed selection include nutritional composition (e.g., energy and nitrogen content), secondary compounds, and seed size (Hoshizaki & Miguchi, 2005; Jensen, 1985; Vander Wall, 2003; Wang et al., 2012; Wang & Ives, 2017). Tannins, a type of secondary compound, are particularly important in selection because they exhibit chemical defense mechanisms against rodents (Pyare et al., 1993; Smallwood & Peters, 1986; Wang & Chen, 2008). Furthermore, the surrounding environment (i.e., vegetation coverage) is thought to influence rodent foraging patterns (Best et al., 2023; Kikuzawa, 1988). Another factor to consider is the presence of other food sources. Forget (1992) and Shimada (2001) showed that foraging methods shift concurrently depending on the simultaneous presence of other food sources.

**FIGURE 1:**
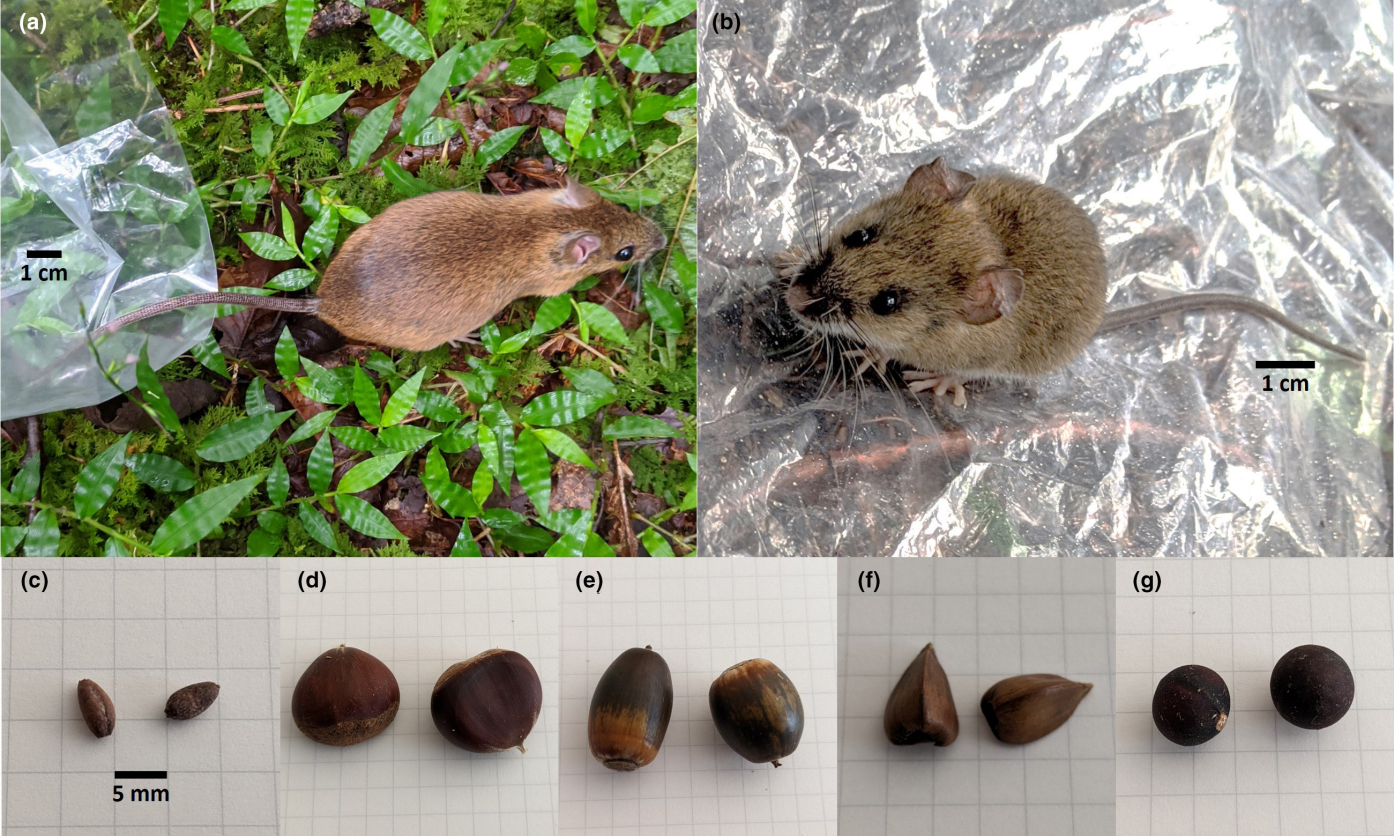
(a) *Apodemus speciosus* (80–140 mm in body length (Kaneko, 2005)), (b) *Apodemus argenteus* (65–100 mm in body length (Kaneko, 2005)), (c) *Sasa borealis* seeds, (d) chestnuts of *Castanea crenata,* (e) acorns of *Quercus crispula*, (f) beechnuts of *Fagus crenata*, and (g) seeds of *Lindera triloba*. One side of each square in the photographs (c–g) is 5 mm long.

Consequently, in this study, we conducted field‐feeding tests using seeds of *S. borealis* (Figure 1c), a dwarf bamboo species, and four other tree species (Figure 1d‐g) with various characteristics in a temperate forest where dead culms of *S. borealis* remained 5 years after mast seeding. In forest areas where the forest floor is covered by dwarf bamboo, tree regeneration is hindered due to its direct impact. Therefore, after the disappearance of dwarf bamboo by mast seeding, tree renewal is expected to proceed. To clarify this cycle, the apparent competition between the seeds must be determined. In a previous study (Suzuki & Kajimura, 2023) conducted at the same site, only *S. borealis* seeds were tested in the forest to identify the foraging species. The results showed that field mice, the large Japanese field mouse *Apodemus speciosus* (Temminck, 1844) (Figure 1a) and the small Japanese field mouse *A. argenteus* (Temminck, 1844) (Figure 1b) forage using predation and removal (including caching). Based on these findings, this study aimed to determine the preference of seed predators for *S. borealis* seeds over tree seeds based on foraging rate and order. The impact of the mast seeding of *S. borealis* on the forest is also discussed.

## 2 MATERIALS AND METHODS

### 2.1 Survey site

The study was conducted in the Takatokke district of Nagoya University Forest (in the Inabu Field affiliated with the Graduate School of Bioagricultural Sciences, Nagoya University), located in the northeastern part of Aichi Prefecture in central Japan. The elevation is approximately 1075 m asl. In 2021, the annual precipitation was 2766 mm, and the average annual temperature was 9.3°C. The forest features a patchy distribution of broad‐leaved forest containing nut‐producing trees such as *Quercus crispula* Blume var. *crispula*, *Castanea crenata* Siebold et Zucc., and *Fagus crenata* Blume and coniferous forest containing conifers such as *Larix kaempferi* (Lamb.) Carrière, *Cryptomeria japonica* (L.f.) D. Don, and *Chamaecyparis obtusa* (Siebold et Zucc.) Endl. At and around this site, the mast seeding of *S. borealis* occurred in 2016 and 2017. We established two plots: Plot‐BF (broad‐leaved forest, 35°13′04′′ N, 137°34′28′′ E) in a secondary forest comprising deciduous broad‐leaved trees (mainly *Q. crispula*, *Cerasus jamasakura* (Siebold ex Koidz.) H.Ohba, and *Lindera triloba* (Siebold et Zucc.) Blume), and Plot‐CF (coniferous forest, 35°12′53′′ N, 137°34′20′′ E) in a forest comprising 60‐year‐old *L. kaempferi* with *L. triloba* in the shrub layer. In each plot, two test points were prepared outside (OUT) and inside (IN) the dead culm cluster of *S. borealis*, approximately 10 m apart.

### 2.2 Feeding test

Chestnuts of *C. crenata* (Figure 1d), acorns of *Q. crispula* (Figure 1e), beechnuts of *F. crenata* (Figure 1f), and seeds of *S. borealis* and *L. triloba* (Figure 1g) were used for the feeding tests. All seeds, including chestnuts, acorns, and beechnuts, are hereafter referred to as “seeds”. The seeds of these four tree species selected for this study were obtained from the surrounding forest area. They exhibited evidence of rodent foraging and were readily available. *S. borealis* seeds were harvested in 2017 in Dando National Forest, Aichi Prefecture, threshed, and stored at room temperature (20–25°C) in our laboratory until required for further testing. The seeds of *C. crenata*, *Q. crispula*, *F. crenata*, and *L. triloba* were collected around the plot in 2022. Weight and detailed information of each seed are provided in Table 1. Seeds used in the tests were selected from sound seeds free from insect damage. Preferences among seeds were made by simultaneously comparing each tree seed with *S. borealis* seeds. For the tests of *C. crenata* or *Q. crispula*, a set comprised 5 g of *S. borealis* seeds and four seeds of *C. crenata* or *Q. crispula*. For the tests of *F. crenata* or *L. triloba*, a set comprised 2.5 g of *S. borealis* seeds and two seeds of *F. crenata* or *L. triloba*. These ratios of masses and numbers were based on our evidence and observation of a previous study that exclusively used *S. borealis* (Suzuki & Kajimura, 2023). We determined the amount of *S. borealis* required for approximate seed consumption in one night and the number of tree seeds available on the forest floor. Each tree seed was weighed individually before the test.

**TABLE 1:** Traits of seeds used in the study.

	Type	Seeding cycle	Carbohydrate level [%]	Energy [kJ/g]	Secondary compound	Seed size [±SD, g] (*n*) [G]	Coat thickness
*Sasa borealis*	Seed	120 years	76.6 [A]	14.9 [A]	–	0.0268 ± 0.0048, (*50*)	Thin
*Castanea crenata*	Chestnut	1 year	36.9 [B]	6.9 [B]	–	3.0660 ± 1.1053, (*80*)	Thick
*Quercus crispula*	Acorn	2–3 years [C]	90.5 [D]	14.3 [D]	Tannins [E]	2.8188 ± 0.7415, (*80*)	Thick
*Fagus crenata*	Beechnut	5–7 years [F]	21.9 [D]	22.1 [D]	–	0.1270 ± 0.0319, (*40*)	Thick
*Lindera triloba*	Seed	1 year	–	–	–	0.3590 ± 0.1048, (*40*)	Thick

References: [A] Shimada et al., 2019, [B] MEXT, 2015, [C] Saitoh et al., 2007, [D] Hoshizaki, 2009, [E] Shimada & Saitoh, 2003, [F] Suzuki et al., 2005, [G] Measured value in this study.

**FIGURE 2:**
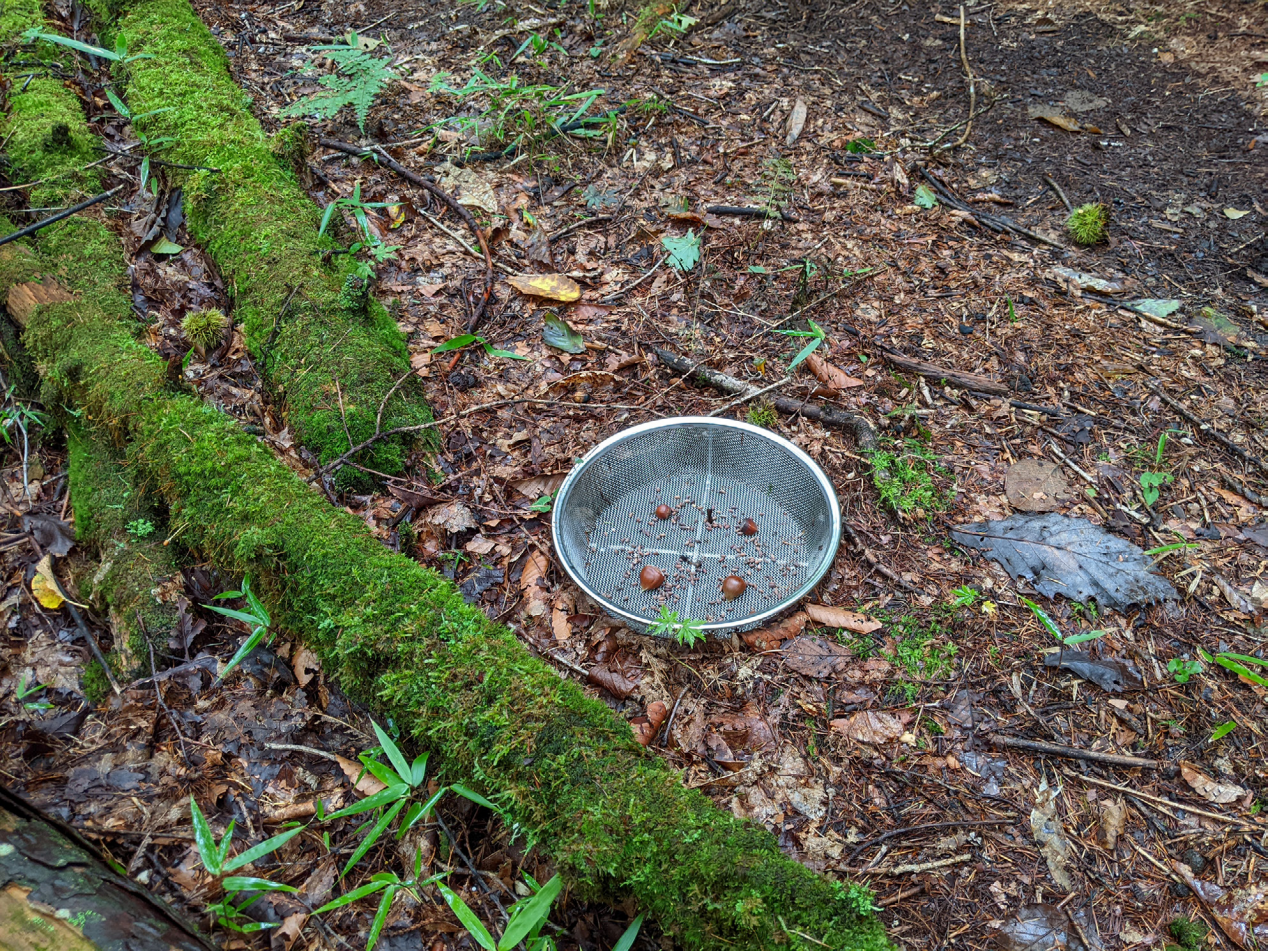
A food station (container setting up *Sasa borealis* and *Castanea crenata* seeds) in the forest (Plot‐BF‐OUT, 2022/9/15).

At each of the four test points (Plot‐BF‐IN, Plot‐BF‐OUT, Plot‐CF‐IN, and Plot‐CF‐OUT), a food station was established by positioning a shallow metal container (22 cm in diameter) on the ground (Figure 2). Seed sets were then placed at these food stations at each test point. The tree seeds to be tested were identified by weight and placed individually at the same test point. The following day, all the remaining seeds were collected.

The remaining *S. borealis* seeds were weighed. The weight of seeds utilized by the visiting animals was calculated by subtracting the remaining weight from the initial weight (5 or 2.5 g). Additionally, the remaining tree seeds were counted. The seed foraging rate was defined as the percentage of lost seeds (or weight) to initial seeds (or weight). A series of feeding tests were conducted from September 15 to November 16, 2022. To align with the timing of tree seed drop, *C. crenata* and *Q. crispula* tests were performed from September to early October (i.e., early fall), whereas *F. crenata* and *L. triloba* tests were performed from late October to November (i.e., late fall). Five tests were repeated for each tree seed (four species) at each point (four locations).

A trail camera (HYKE HCSP2; Hyke Inc., Hokkaido, Japan) was placed approximately 50 cm away from each container to monitor its contents and surroundings. The camera was set to high sensitivity to record a video after the sensor response and to capture an hourly time‐lapse photograph. The videos were analyzed to identify the species of visiting animals and their seed utilization behaviors (predation or removal) at each food station. However, the videos did not permit the distinct identification of individual animals. The foraging rate and order were used to determine seed preference. In addition, a preference for seed size within the same tree species was analyzed. No permissions were required prior to conducting this research, as only automatic recording of naturally visiting animals was used.

### 2.3 Statistical analyses

The differences in the foraging rate after one night between *S. borealis* and each tree species were compared using the Mann–Whitney *U*‐test. To analyze the effects on the foraging rate of *S. borealis*, a generalized linear model was used, including season (early fall or late fall), plot (Plot‐BF or Plot‐CF), test point (IN or OUT), and tree species tested at the same time as explanatory variables. The preference of field mice in terms of foraging order was analyzed in two ways: whether *S. borealis* or tree seeds were foraged first in each test and the percentage of mice that foraged on *S. borealis* or tree seeds until all seeds of either type were gone. The latter was analyzed separately for *A. speciosus* and *A. argenteus* during the tree seed tests, and differences between the two were compared using Fisher's exact test. Comparisons of size (and weight) with tree seed foraging order were conducted using Student's *t‐*test or Tukey's honestly significant difference test. All analysis programs were run in R v4.1.2 (R Core Team, 2021).

## 3 RESULTS

### 3.1 Visiting animals and their behaviors

In 80 tests, five different mammal species visited the food containers. The most frequent visitors were field mice of two species: *A. speciosus* (692 times) and *A. argenteus* (486 times). Smith's red‐backed vole *Eothenomys smithii* (Thomas, 1905), raccoon dog *Nyctereutes procyonoides* (Gray, 1834), and Japanese weasel *Mustela itatsi* (Temminck, 1988) also visited the area multiple times. *S. borealis* seeds were foraged by *A. speciosus*, *A. argenteus*, *E. smithii* (Video S1), and *N. procyonoides* (Video S2). However, *E. smithii* only preyed 13 times, and *N. procyonoides* preyed only 4 times. Therefore, we only considered foraging due to *A. speciosus* and *A. argenteus*. Tree seeds were exclusively foraged by *A. speciosus* (Video S3) and *A. argenteus* (Video S4). *M. itatsi* only sniffed the inside of the container. In addition, no birds visited the containers, although camel crickets (Rhaphidophoridae) were observed moving *S. borealis* on several occasions.

Both predation (consumed in situ) and removal (removed to somewhere in the mouth) from the container were observed among *A. speciosus* and *A. argenteus*, whereas *E. smithii* was only observed removing, and *N. procyonoides* was only observed predating. *S. borealis* seeds were both consumed in situ and removed. As for tree seeds, all were removed except for one *C. crenata* seed that was partially consumed in situ. It was also observed that *A. speciosus* and *A. argenteus* carried several seeds at a time during the removal of *S. borealis*.

### 3.2 Foraging rate of seed species

**FIGURE 3:**
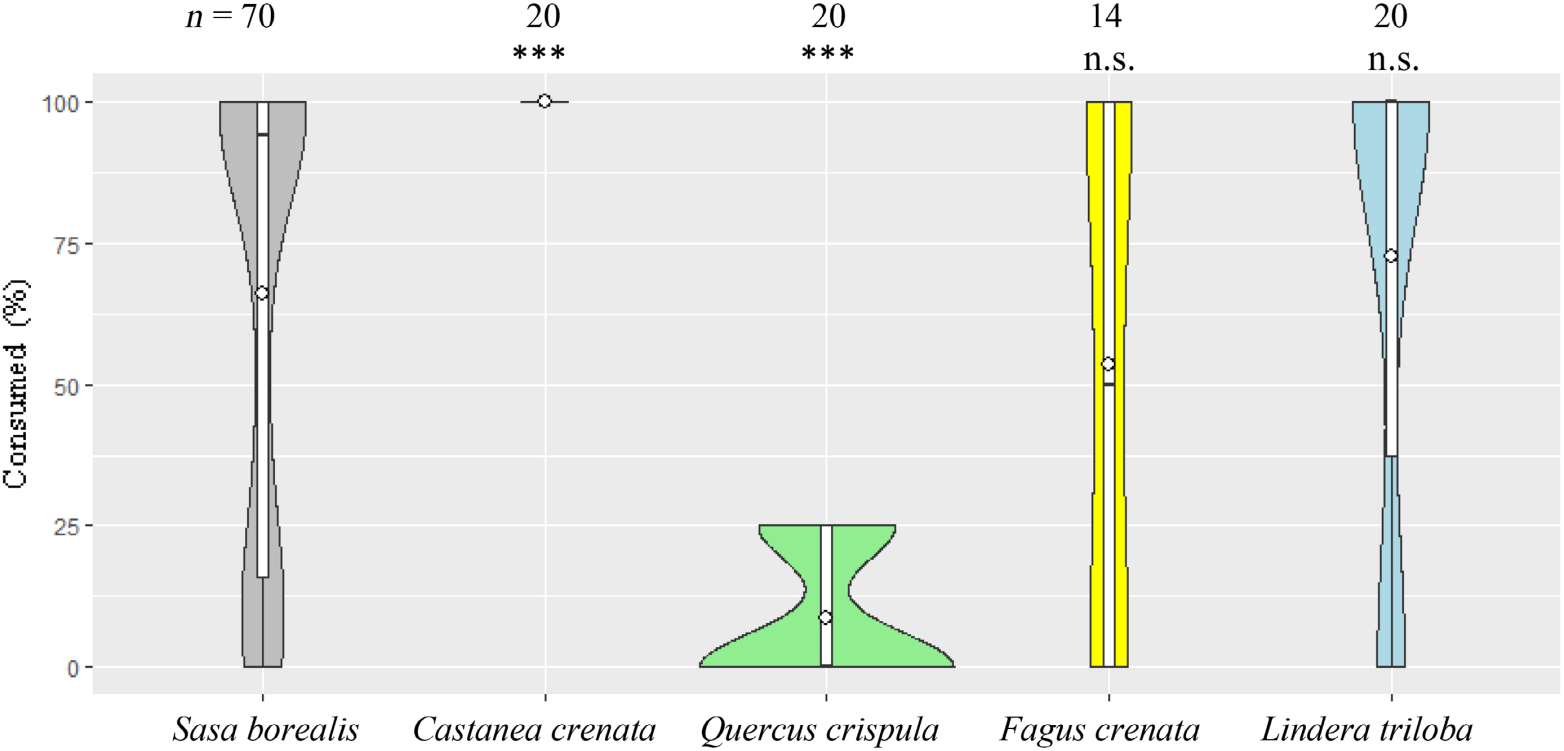
Violin plot representing the distribution of the foraging rate of each seed species. The white circles in the box plots indicate the mean. ***: statistically significant at *p* < .005 using the Mann–Whitney *U*‐test with *S. borealis*.

The foraging rates of each seed species after one night of testing are shown in Figure 3. For *F. crenata*, some sterile seeds that could not be determined from the outside view were included. This is because *F. crenata* nuts grow regardless of whether the embryo matures or not (Nakashizuka, 2009). Therefore, the remaining seeds were collected and inspected for contents, and the six tests in which only sterile seeds were tested were excluded from the results. The mean (±SD) for each species was 66.1 ± 41.0% (*n* = 70) for *S. borealis*, 100% (*n* = 20) for *C. crenata*, 8.8 ± 11.9% (*n* = 20) for *Q. crispula*, 53.6 ± 44.3% (*n* = 14) for *F. crenata*, and 72.5 ± 43.2% (*n* = 20) for *L. triloba*. The foraging rate of *C. crenata* was significantly high, and that of *Q. crispula* was significantly low (Mann–Whitney *U*‐test, *p <* .005).

The generalized linear model analysis revealed that the plot and testing with *C. crenata* were significantly related to the foraging rate of *S. borealis* (Table 2). Plot‐CF was found to have a higher foraging rate than Plot‐BF, and the foraging rate was also higher when tested with *C. crenata* at the same time. Microenvironmental differences, such as foraging outside or inside of dead culms, did not influence the foraging rates.

**TABLE 2:** Result of general linear model analysis for the foraging rate of *S. borealis.*

Explanatory variable	Estimate	Std. error	*t*‐Value	*p*
(Intercept)	29.314	7.865	3.727	.000412***
Season	7.575	8.842	0.857	.39483
Plot	58.752	6.81	8.627	2.6E‐12***
Test point	−10.065	6.684	−1.506	.137053
*Castanea crenata*	20.827	9.379	2.221	.029922*
*Quercus crispula*	NA	NA	NA	NA
*Fagus crenata*	7.359	9.852	0.747	.457854
*Lindera triloba*	NA	NA	NA	NA

* Statistically significant at *p* < .05.

*** Statistically significant at *p* < .001.

### 3.3 Foraging order

**FIGURE 4:**
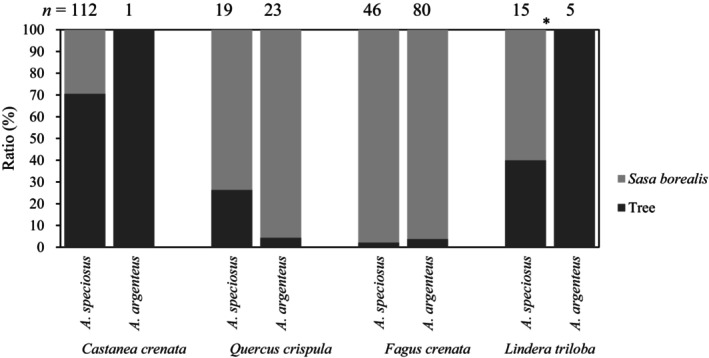
Percentage of observation frequency of which seed of *S. borealis* or trees was selected by two species of field mice until either seed was gone or the next morning, whichever occurred first. *Statistically significant at *p* < .05 using Fisher's exact test.

Based on the camera images of *S. borealis* and tree seeds, the first to forage was *C. crenata* in 20 of 20 trials (100%), *L. triloba* in 11 of 18 trials (71%), *F. crenata* in 2 of 12 trials (16%), and *Q. crispula* in 2 of 20 trials (10%). These results excluded tests in which there was a camera malfunction. In addition, Figure 4 shows whether the two species of field mice were selected for *S. borealis* seeds or tree seeds until either seed was gone or until the next morning. The ratio represents the percentage of times each seed was selected out of the total number of foraging behaviors during all the trials. For *A. speciosus*, the foraging of *C. crenata* was higher (71%) than that of *S. borealis*. *L. triloba* foraging was lower (40%) than *S. borealis* foraging but higher than *Q. crispula* and *F. crenata* foraging. As for *A. argenteus*, only one foraging instance was observed in *C. crenata* tests, but *C. crenata* was foraged, and in *L. triloba* tests, 100% of the foraging was for *L. triloba*. In contrast, in the *Q. crispula* and *F. crenata* tests, *S. borealis* was foraged more than 95% of the time. The *L. triloba* tests showed significant differences between *A. speciosus* and *A. argenteus* (Fisher's exact test, *p* < .05).

**FIGURE 5:**
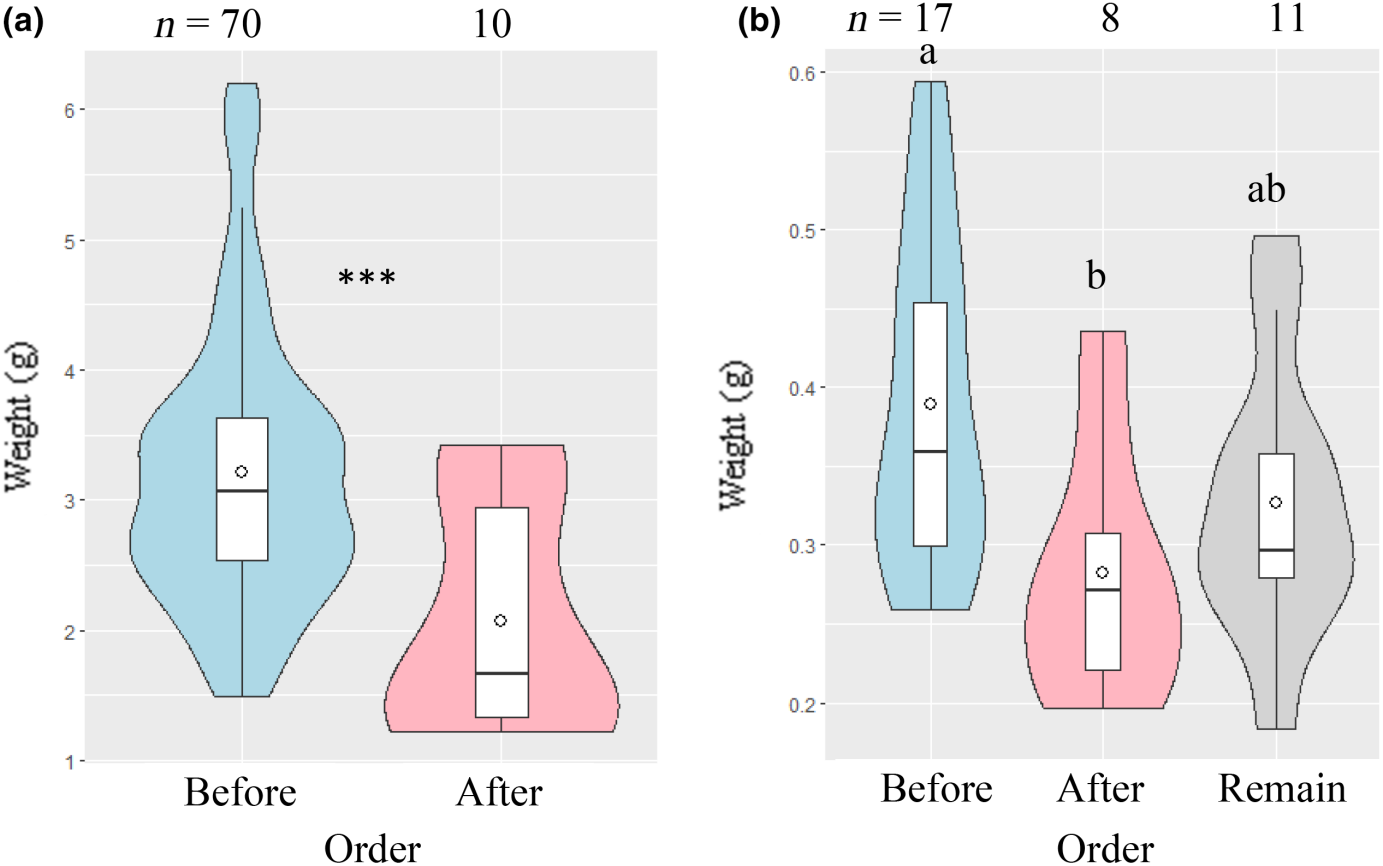
Violin plots representing the weights of seeds foraged before *Sasa borealis*, seeds foraged after *S. borealis*, and remaining seeds. (a) *Castanea crenata* and (b) *Lindera triloba*. The white circles in the box plots indicate the mean. ***Statistically significant at *p* < .005 using Student's *t*‐test. Different letters indicate significant differences between seeds at *p* < .05 using Tukey's honestly significant difference test.

*C. crenata* and *L. triloba*, the most foraged species, were compared by the difference in weight between seeds foraged before *S. borealis*, seeds foraged after *S. borealis*, and seeds left behind (Figure 5). For both species, seeds foraged before *S. borealis* were significantly heavier than those foraged after *S. borealis* (*C. crenata*: Student's *t‐*test, *p* < .005; *L. triloba*: Tukey's honestly significant difference test, *p* < .05).

## 4 DISCUSSION

### 4.1 Visitors and seed foragers

Two species of field mice, *A. speciosus* and *A. argenteus*, were the most abundant foragers of seeds. This result is consistent with the fact that these two species of field mice mainly forage on seeds (Kaneko, 2005). Then, foraging for *S. borealis* seeds was the same as that reported previously (Suzuki & Kajimura, 2023). These results support the hypothesis that the increase in their population after the mast seeding of *S. borealis* was due to increased food resources (Suzuki et al., 2022). In addition, foraging of *S. borealis* seeds by *E. smithii*, another rodent species, and *N. procyonoides* of the family Canidae, was newly identified. According to the observed behavior, *E. smithii* consumes the green part of plants and the starch of seeds (Kaneko, 2005). *N. procyonoides* is omnivorous and highly adaptable; it feeds on cereal, including Triticeae (Elmeros et al., 2018). Similarly, *N. procyonoides* was newly found to feed upon *S. borealis* of Poaceae on some occasions. The observation that neither of these two species was seen foraging in a previous study (Suzuki & Kajimura, 2023) may be attributed to a low encounter rate. *M. itatsi* was seen sniffing the container, possibly lured by rodents, its primary food source (Kaneko et al., 2013). Camel crickets forage *S. borealis* seeds when fed in rearing (H. Suzuki, H. Kajimura, unpublished data), and we consider the movements recorded on camera as part of a foraging behavior sequence.

Regarding the caching behavior exhibited by rodents, particularly *A. speciosus* and *A. argenteus*, both predation and removal behaviors were observed for *S. borealis*, while all tree seeds except for one *C. crenata* were removed. Rodent behavior can vary depending on whether they remove or predate on prey seeds, which is often influenced by the seed size (Vander Wall, 2003; Wang et al., 2012). In this experiment, *S. borealis* seeds averaging 0.0268 g were consumed in situ. However, *F. crenata* seeds averaging 0.1270 g or more, were removed, indicating their division for dietary use, as concluded in other seed studies (Vander Wall, 2003; Wang et al., 2012).

The results of the generalized linear model analysis for the foraging rate of *S. borealis* suggested that the factors affecting the foraging rate of *S. borealis* were the plot and the presence or absence of *C. crenata*. The higher foraging rate of *S. borealis* in Plot‐CF aligns with that reported previously by Suzuki and Kajimura (2023), suggesting that Plot‐CF may have fewer alternative food sources due to the specific characteristics of the forest area. The same plots were used for all tests; thus, the plot does not influence the preference intensity between the seeds. In contrast, the presence of *C. crenata* increased the foraging rate for *S. borealis*, suggesting that *C. crenata* may have been an attractant for mice. Although *C. crenata* should be considered for preference intensity between the simultaneously tested seeds, we consider that *S. borealis* was selected with the same preference throughout the experiment for the following discussion.

### 4.2 Seed preference factors of field mice

Regarding the seed preference of field mice, the overnight foraging rate results (Figure 3) suggested that *C. crenata* may have the highest preference, followed by *L. triloba*, *S. borealis*, and *F. crenata* at similar levels, and *Q. crispula* at the lowest level. Preference was then judged in terms of the foraging order. The results of the first seeds foraged by mice showed that *C. crenata* and *L. triloba* had a higher foraging percentage before *S. borealis*, indicating a stronger preference. *Q. crispula* and *F. crenata* were considered less preferential compared with *S. borealis*, as the percentage of *S. borealis* foraging was initially higher than that of *Q. crispula* and *F. crenata*. In addition, the percentage of rodents foraging on *S. borealis* seeds versus tree seeds by the time the tree seeds were fully foraged or the next morning (Figure 4) also suggests a high preference for *C. crenata* and *L. triloba* and a low preference for *Q. crispula* and *F. crenata*. The two viewpoints on foraging rate and foraging order indicated by the results suggest that the seed preference of mice is *C. crenata*, *L. triloba*, *S. borealis*, *F. crenata*, and lastly, *Q. crispula*.

*S. borealis* has a high carbohydrate level of 76.6% (*C. crenata*, 36.9%; *Q. crispula*, 90.5%; and *F. crenata*, 21.9%) (Hoshizaki, 2009; MEXT, 2015; Shimada et al., 2019). The energy of *S. borealis* is 14.9 kJ/g (Shimada et al., 2019). Compared with *C. crenata* (6.9 kJ/g), *Q. crispula* (14.3 kJ/g), and *F. crenata* (22.1 kJ/g), the energy content of *S. borealis* is not as high as that of *F. crenata* but is sufficient (Hoshizaki, 2009; MEXT, 2015). High carbohydrates and energy content are also observed in seeds of other bamboo species (Kumawat et al., 2014). Since *S. borealis* seeds are smaller than tree seeds, foragers require a larger number to ingest, but its large supply also makes it a more valuable food source.

*C. crenata*, which was the most preferred species, has been reported as a food source and cache target for several animals, particularly *Apodemus* spp. (Seiwa et al., 2002; Tanikawa, 2009). *C. crenata* has a lower energy content obtained per gram than *S. borealis*, but its larger seed size makes it preferred for caching and may result in a greater benefit.

*Q. crispula* is a large seed like *C. crenata* but is high in tannins and low in fat and proteins; thus, it has low value as a forage resource (Shimada & Saitoh, 2003, 2006). However, field experiments using *Q. crispula* seeds have revealed caching behavior by *A. speciosus* and *A. argenteus*, and *Q. crispula* was detected in the diet analysis of both species; thus, *Q. crispula* is utilized as a food source (Miura & Okitsu, 2006; Sato et al., 2018). Additionally, Saitoh et al. (2007) showed that *A. speciosus* population dynamics occur in response to *Q. crispula* abundance, whereas Hoshizaki and Miguchi (2005) noted that the relationship is unclear. Therefore, it is likely that environmental factors, such as the availability of other food sources, impact the value of *Q. crispula* utilization. In this experiment, the presence of another food source, i.e., *S. borealis*, may have more remarkably reduced the utilization value of *Q. crispula*.

*F. crenata* contains little defensive substance (Nakashizuka, 2009), is rich in fat, and is a high‐quality food for mice (Hashizume, 1979; Hoshizaki, 2009; Sugawara, 1972). *F. crenata* is strongly associated with mice, and the population dynamics of *Apodemus* spp., particularly *A. speciosus*, have been reported to occur in response to its abundance (Hoshizaki & Miguchi, 2005; Miguchi, 1988). In this experiment, the preference for *F. crenata* was found to be similar to that of *S. borealis* in terms of foraging rate, but the preference was lower than that of *S. borealis* in terms of foraging order. This difference may be due to the ease of carrying and eating the seeds. Each *F. crenata* seed was larger than that of *S. borealis*, and mice could carry multiple *S. borealis* seeds when removing them, whereas only one *F. crenata* seed was carried at a time. Li et al. (2018) found a relationship between seed preference and husk peel ability through a field experiment in which several species of seeds were used. *F. crenata* is covered by a hard shell, whereas *S. borealis* has an easily peeled chaff. Therefore, *S*. *borealis* may have a higher preference because it is more efficient to forage.

The higher preference for *L. triloba* compared with *S. borealis* is difficult to determine because the seeds have not been studied sufficiently, and their characteristics are unknown. However, *Lindera melissifolia* of the same genus is known to have a higher ratio of saturated acid to unsaturated acid compared with other tree seeds, such as *Quercus* spp. (Connor et al., 2007). Mice have been reported to forage for the seeds of *L. melissifolia* when tested in the field (Martins et al., 2015). Therefore, it is likely that *L. triloba* also contains some elements favored by mice.

Regarding preference for *L. triloba* among field mouse species, *A. argenteus* selected 100% *L. triloba*, indicating a significantly stronger preference than *A. speciosus* for *L. triloba*. Similarly, *A. speciosus* clearly foraged more for *Q. crispula* than *A. argenteus*. *A. speciosus* has a higher tannin tolerance (Shimada et al., 2004). Sato et al. (2018) found that both *Apodemus* species forage for *Q. crispula*, but *A. speciosus* has a higher percentage of *Q. crispula* in their diet analysis, which is also in accordance with our results.

There was a significant difference in the size of *C. crenata* and *L. triloba* seeds foraged before and after *S. borealis* (Figure 5), clearly indicating that larger seeds are preferred. For example, Wang et al. (2012) showed that there was a preference for larger acorns among the same species in a seed‐feeding experiment. In this experiment, the degree of preference was much more apparent when tested with *S. borealis*.

## 5 CONCLUSIONS

This study, conducted over a brief period of approximately 3 months, coinciding with each seed supply timing, yielded the first data set identifying seed foragers and their preferences.

Our experiments were designed to compare *S. borealis* seeds with individual tree species for their reliable identification from video images. However, simultaneous testing of *S. borealis* with multiple tree species would better match field conditions (flora and its distribution), thereby determining more realistic seed preferences by mouse species. We plan to develop a better experimental system in the future.

Based on the reported results of mice preference, potential interactions between trees and *S. borealis* in forest ecosystems regarding seed foraging pressure were considered. *S. borealis* seeds in forests with *C. crenata* may have higher foraging rates and lower seedling establishment rates than those without. If the 120‐year cycle of *S. borealis* seeding coincides with rich harvests of *Q. crispula* or *F. crenata*, the predation pressure may be reduced, increasing the establishment rate of these tree seedlings. This relationship is assumed to eventually lead to inhibited regeneration owing to the cover of dwarf bamboo, and future follow‐up investigations related to the regeneration of the forests may be required.

## AUTHOR CONTRIBUTIONS

**Hanami Suzuki:** Conceptualization (lead); data curation (lead); formal analysis (lead); funding acquisition (equal); investigation (lead); methodology (lead); visualization (lead); writing – original draft (lead). **Hisashi Kajimura:** Conceptualization (supporting); funding acquisition (equal); methodology (supporting); project administration (lead); resources (lead); supervision (lead); validation (lead); writing – review and editing (lead).

## CONFLICT OF INTEREST STATEMENT

The authors declare that they have no competing interests.

## Supporting information


Video S1.

Video S2.

Video S3.

Video S4.

## Data Availability

The datasets analyzed for this study can be found on Figshare https://figshare.com/s/3c7ae682731c3bb66b62.
